# Gut phages and their interactions with bacterial and mammalian hosts

**DOI:** 10.1128/jb.00428-24

**Published:** 2025-01-23

**Authors:** Marshall Godsil, Nathaniel L. Ritz, Siddarth Venkatesh, Alexander J. Meeske

**Affiliations:** 1 Department of Microbiology, University of Washington 312771 , Seattle, Washington, USA; 2 Institute for Systems Biology 7268 , Seattle, Washington, USA; University of California San Francisco, San Francisco, California, USA

**Keywords:** phages, microbiome

## Abstract

The mammalian gut microbiome is a dense and diverse community of microorganisms that reside in the distal gastrointestinal tract. In recent decades, the bacterial members of the gut microbiome have been the subject of intense research. Less well studied is the large community of bacteriophages that reside in the gut, which number in the billions of viral particles per gram of feces, and consist of considerable unknown viral “dark matter.” This community of gut-residing bacteriophages, called the gut “phageome,” plays a central role in the gut microbiome through predation and transformation of native gut bacteria, and through interactions with their mammalian hosts. In this review, we will summarize what is known about the composition and origins of the gut phageome, as well as its role in microbiome homeostasis and host health. Furthermore, we will outline the interactions of gut phages with their bacterial and mammalian hosts, and plot a course for the mechanistic study of these systems.

## INTRODUCTION

The idea that our bodies are home to abundant microbial life began when Antonie van Leeuwenhoek first peered at his dental scrapings through a microscope in the late 17th century (1). Since those early days, researchers have sampled every surface and cavity of the human body, characterizing the microbes that live there (2, 3). We now have an appreciation for the diversity of human**-**associated microbes, their unique distribution through the body, and their outsized effect on human health. The densest population of human-associated microbes resides in the distal gut and is collectively referred to as the gut microbiota. The gut microbiota is made of bacteria, fungi, archaea, and viruses, yet the bulk of research to date has focused on the bacterial constituent. Gut viruses are among the most abundant biological entities in the human body (4), but anywhere from 75% to 99% of gut viral metagenomic reads do not align to any known genome (5). Although many members of the gut virome remain uncultured and uncharacterized, the role of gut viruses in human health is gaining increasing recognition. The focus of this review are members of the virome that infect gut bacteria, individually known as gut bacteriophages and collectively termed the gut phageome.

**Fig 1:**
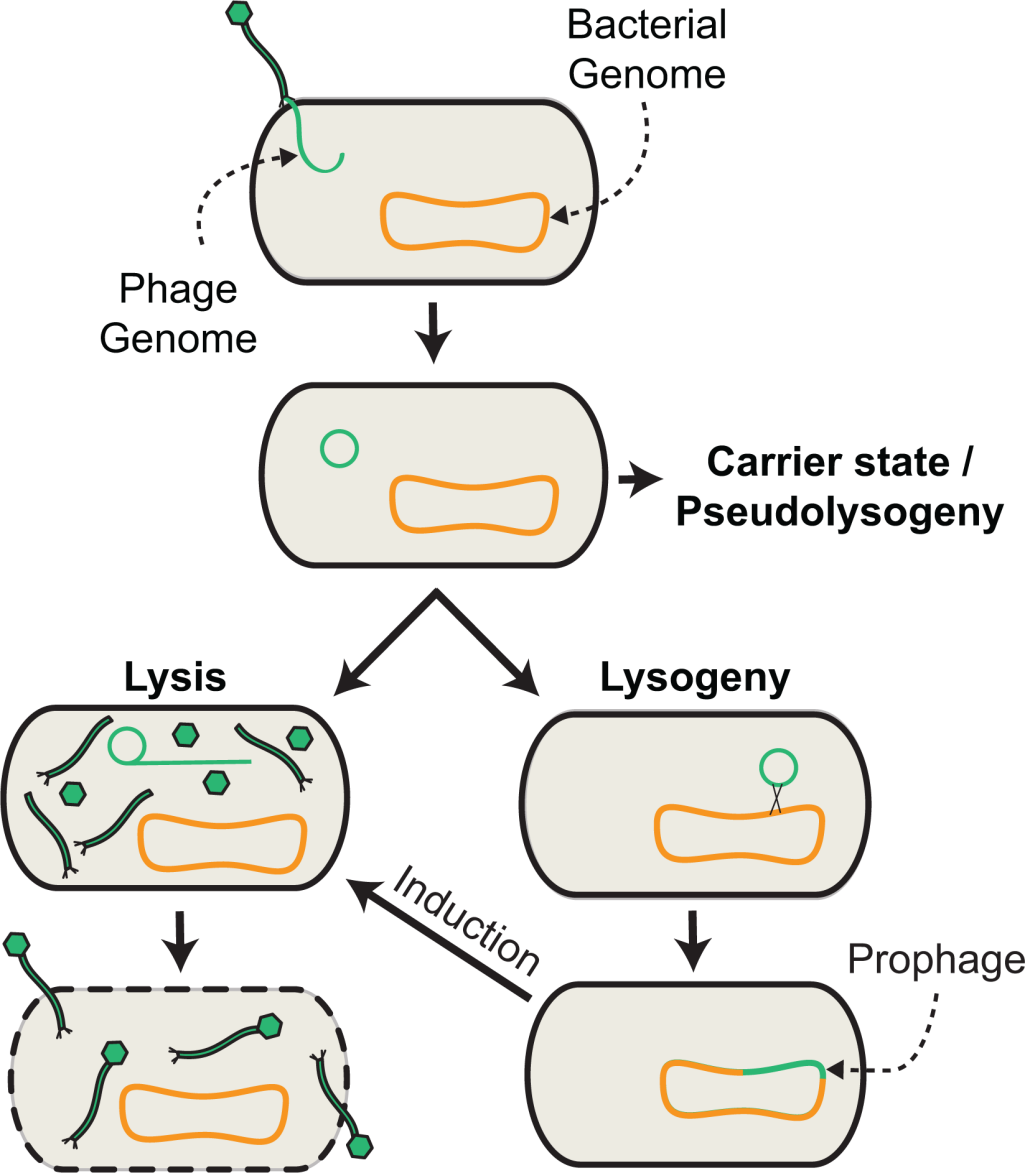
Phage lifestyles. Depicted are various forms of phage replication. Phages initially adsorb to surface structures on their bacterial hosts, triggering injection of their genome as a linear molecule into the host cell cytosol. After genome circularization, diverse phages pursue different lifestyles. Under certain conditions, some phages are maintained episomally in various carrier states that do not result in cell lysis. Both virulent and temperate phages participate in lytic replication, wherein phage genomes replicate and are packaged into newly synthesized viral particles, which are released by cell lysis. Temperate phages participate in lysogeny, where the phage genome recombines with the bacterial genome, resulting in prophage integration. Integrated prophages largely silence their lytic genes but can influence bacterial physiology by expressing other phage-encoded factors or re-wiring host transcription.

The gut microbiota influences many aspects of human health: from maintaining normal metabolism, to neural development, to the mounting of an effective immune response (6–8). Numerous discoveries linking the gut microbiota to human health have emerged in recent decades. Yet, this revolution in our understanding of the gut began in the early 20th century. At that time, researchers viewed the microbiota as a harmful vestigial organ teeming with toxin-producing bacteria that required inhibition. While these claims were not borne out, they correctly identified an intimate link between the microbiota and human health. At the same time, the study of bacteriophages was also entering its infancy (9). Bacteriophages, or phages for short, are viruses that propagate in bacterial hosts, often killing host cells upon release. Some phages exhibit non-lethal modes of infection, influencing bacterial physiology through the expression of prophage-encoded genes, the re-wiring of bacterial gene transcription, and the transfer of novel genetic material (10–13) (Fig. 1). Through both lysis-mediated killing and non-lytic conversion of their hosts, bacteriophages shape bacterial communities. While the effects of phage on bacterial populations have been well explored in aquatic and terrestrial environments (14), only recently has focus turned to their interactions with the gut microbiota. Given the strong selective pressures that phages exert on bacteria in most environments and the crucial role of the gut microbiota in human health and disease, the biology of gut phages has garnered great interest in the last decade.

Despite growing interest in the gut phageome, tools to culture and study gut phages *in vitro* remain limited. In their absence, high-throughput metagenomic sequencing tools have proved crucial in defining composition of the gut phage community. By comparing longitudinal phageome content between individuals, associations have been established between gut phage communities and different environmental exposures or states of disease. In this review, we will broadly summarize what these findings reveal about the origins and composition of the gut phageome, as well as its relevance to human health. We will also discuss in detail how gut phages interact with their bacterial and mammalian hosts, and some methods for mechanistic investigation of these systems.

## ORIGINS AND COMPOSITION OF THE GUT PHAGEOME

During birth, infants are exposed to non-sterile environments, and the development of their microbiota begins (15). The early gut phageome is characterized by high rates of turnover as scores of bacteria and phages move through the unsettled landscape of the infant gut (16–18). The first phages that successfully colonize the gut are prophages that hitchhike within the chromosomes of early bacterial colonizers (19, 20). These organisms originate from the body and milk of the infant’s mother as well as the surrounding environment, although the mechanisms of strain acquisition are poorly understood. This early microbiota assembly is affected by delivery mode, diet, and antibiotic usage (21–23). Many of the early prophage-harboring colonists are of the phyla Pseudomonadota (previously Proteobacteria), which are prevalent during early life but are eventually displaced by Bacteroidota (previously Bacteroidetes) and Bacillota (previously Firmicutes). These prophages likely re-enter a lytic state in the gut to form the bulk of released viral particles found in stool. The paradigm of prophages as first colonizers makes intuitive sense, as lone phage particles may not find a suitable host to replicate on in a sparsely colonized gut. This model raises interesting questions about whether prophage reactivation is internally coordinated or responds to external signals in the gut. It has been shown previously that members of the microbiota produce toxins, which can trigger prophage induction (24). These toxin producers could kill prophage-harboring colonizers through forced induction resulting in lysis. Alternatively, prophage carriers could restrict new bacterial colonizers through their released phage particles. There are likely many signals for prophage reactivation in the gut, but their relevance to microbiota development and homeostasis is not well understood. Furthermore, the role of phages in microbiota development remains undefined. How the developmental trajectories of the gut microbiota and the gut phageome influence each other and human development is an exciting question that is largely unexplored.

In the first days of colonization, the gut phageome is unstable, rapidly increasing in density and diversity (19, 20, 25). By 1 month of age, infant stool contains around 10^9^ viral particles, with more than 98% of viral reads originating from phage (26). As the phageome develops, Siphovirus colonizers are displaced by expanding populations of Myoviruses*, Microviridae,* and eventually *Crassviridae* (Box 1) (16, 27). The formation of the core phageome mirrors the development of the mature microbiome. The cycles of ecological succession and diversification of gut phages ultimately stabilize, and a core phageome forms. The composition of the gut phage community is highly linked to the microbiota, both of which are affected by diet, and a slew of other environmental factors (e.g., antibiotic intake, diet, etc.) (19, 25, 28–31). The developed phageome consists of a temporally stable core of phage taxa and a more variable shell, each of which are highly specific to an individual (26). This core of phage species has been termed the “persistent personal virome” (PPV) and is made up primarily of *Microviridae* and *Crassviridae*. In many individuals, both *Microviridae* and *Crassviridae* viral genomes can remain consistently present at high abundances for periods of 12 months or longer. While stability and slow drift of core viral genomes are more common in the gut, on occasion, strains of core phages are rapidly replaced by emergent variants. *Microviridae* and *Crassviridae* are thought to be mostly virulent gut phages, which may be counterintuitive given their stable association with gut bacteria. This quasi-equilibrium is an enigmatic feature of these virulent gut phages (26, 32). Many PPVs infect common gut symbionts of the Bacteroidota and Bacillota phyla. These phages are temporally stable and highly abundant but make up only ~10% of annotated phage species (33). The more transiently detected phages are of the class Caudoviricetes and family *Inoviridae*. Among both common and variable species are innumerable uncultured phages whose replicative strategies and effects on host bacteria are undefined. Thus, while we are beginning to understand general trends of phageome development, the prevalence of viral “dark matter” leaves much to be discovered about this crucial component of the gut microbiota.

Box 1 **Crassviridae**

Discovered in 2014, *Crassviridae* are the most prevalent dsDNA phages in the human microbiota, present in 73% of human fecal samples (34, 35). These phages are extremely stable in the gut, persisting for years at a time (26). *Crassviridae* primarily infect members of the Bacteroidota phylum (36, 37). Ancient co-evolution of host and phage is evident in the sequence similarity of DNA primases and carbohydrate-binding domains, probably adapted from Bacteroidota by crAssphages. These carbohydrate-binding domains, along with *Crassviridae* tail proteins, are likely necessary for phage entry, are subject to strong positive selection (38), and undergo frequent genetic variation through the action of tail fiber adjacent diversity-generating retroelements (DGRs) (39). Bacteroidota hosts frequently evade phage predation via phase variation of bacterial cell surface components required for phage adsorption (40, 41). Despite this, *Crassviridae* persist with their hosts for extended periods both in the gut and in pure culture (42). While these phages are virulent, i.e., incapable of integration into the bacterial host chromosome, they seem to assume a carrier state (Fig. 3C and D). Recent findings assert that a widespread crAssphage, *Carjvirus communis*, maintains its genome outside of the host chromosome, like the P1 phage-plasmid of *Escherichia coli* (43). This study showed that *C. communis* encodes both plasmid and phage genes, expressing plasmid genes more often than phage genes. Whether this is true of *Crassviridae* broadly remains to be seen, yet these findings could explain their persistence and minimal impact on bacterial hosts. While no direct role in microbiota homeostasis has been identified for *Crassviridae*, they seem to be associated with healthy members of industrialized societies and are depleted in individuals with inflammatory bowel disease (IBD) (32). Undoubtedly, as enthusiasm and cultured representatives of this family continue to grow, so will the understanding of this keystone gut phage.

## ASSOCIATIONS BETWEEN GUT PHAGE COMMUNITY COMPOSITION AND DISEASE

The quest to identify core members of a phageome that are present in healthy individuals and absent in diseased individuals began when Manrique et al. identified 10 phage genomes that were shared by 62% of healthy individuals in a 166-person cohort (44). These core phages are only present in 42% and 54% of patients with ulcerative-colitis (UC) and Crohn’s diseases (CD), respectively. In 2015, one study described an increase in Caudoviricetes taxonomic α-diversity (diversity within an individual) associated with both UC and CD, thus linking gut phage α-diversity to disease (45). Contrary to this, another study found that analyzing the same data set while including unknown viral sequences dissipates this signature of Caudoviricetes α-diversity (32). A complicating thread of these studies is the use of multiple displacement amplification (MDA) sequencing protocols. MDA can result in an overamplification of small circular genomes, minimizing the signal of lower abundance phage genomes. A more recent study, looking strictly at unamplified fecal phageomes, was also unable to associate measures of phageome α-diversity with disease (33). While analysis of bacterial 16s rRNA α-diversity clearly distinguishes healthy and inflammatory bowel disease (IBD) patients, analysis of unamplified phageome α-diversity did not show clear distinctions between populations. This suite of findings emphasizes the importance of consistency and optimization of metagenomics approaches. Yet, the study of unamplified phageomes showed that healthy patient populations were more likely to be composed of shared “communal” viruses, than non-healthy controls, giving some credence to the healthy gut phageome concept.

**Fig 2:**
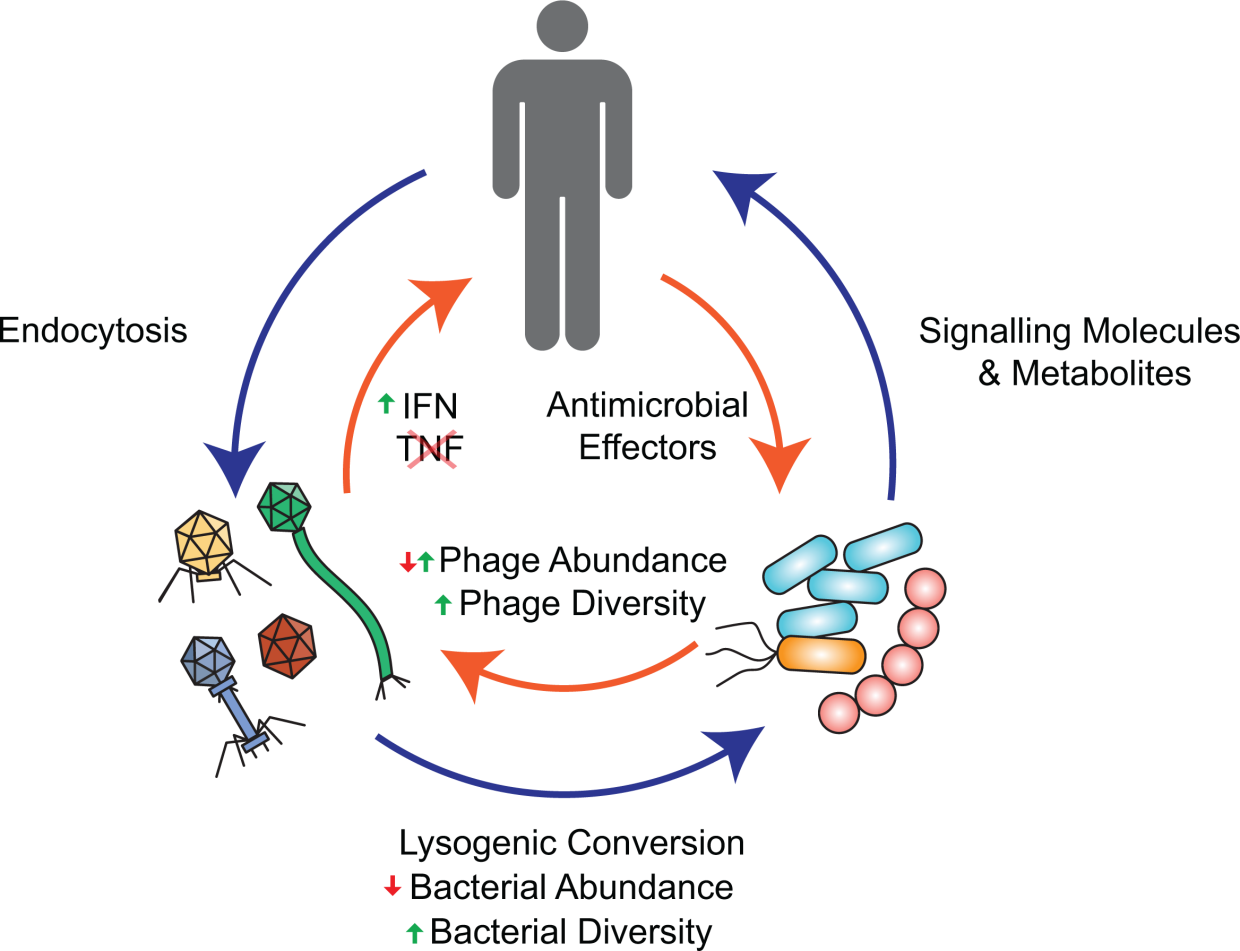
Interactions of phage, bacteria, and human hosts. Depicted are the pairwise interactions of gut phages, their host bacteria, and humans. Gut phages modulate bacterial abundance but can also generate and maintain bacterial diversity through genetic transfer and predatory selection. By acting as a permissive or restrictive growth substrate for phages, gut bacteria affect phage abundance, and in resisting phage infection, bacteria select for diverse phage populations. Following endocytosis, gut phages interact with the mammalian immune system via Toll-like receptor 3 (TLR3) pathways and antagonize anti-bacterial tumor necrosis factor (TNF) signaling via the type I interferon response (IFN). Gut bacteria interface directly with the mammalian immune system through metabolites and signaling molecules including short-chain fatty acids (SCFAs), lipopolysaccharides (LPS), nucleotide oligomerization domain (NOD) ligands, and various other pathways reviewed elsewhere. The mammalian immune system controls gut bacterial populations through antimicrobial peptides (AMPs), soluble immunoglobulin (IgA), and other mechanisms reviewed elsewhere.

More broadly, researchers have also identified decreases in phage α-diversity, which precede the onset of type I diabetes, along with the detection of disease-specific phage contigs (46). Decreases in phage α-diversity were also correlated with obesity and type II diabetes, potentially linking gut phages to both autoimmune and metabolic disorders (47). One disease-associated shift identified in the phageomes of UC and CD patients is a bloom of temperate phage particles. This increase in temperate phages is accompanied by a corresponding decrease in abundance of their hosts, implicating prophage induction as a hallmark of disease. Clooney et al. hypothesized that environmental stressors associated with the inflamed gut, like reactive oxygen species (ROS), induce prophage reactivation, depletion of common microbial species, and activation of the immune system via lysed bacterial contents (32) (Fig. 2). These hypotheses warrant experimental investigation but represent a potential causal link between the life cycle of phages and IBDs. Establishing such a link from case-control phageome studies alone is challenged by an inherent “chicken and egg”-like dilemma. While changes in the phageome might affect the microbiota and cause disease, it is also possible that disease states elicit changes in the microbiota that consequently alter phageome composition. In many cases, the effects of any perturbation on both entities could be inseparable. Attributing disease causality to phageome changes becomes an easier task when experimental models are at hand to evaluate the sufficiency and necessity of phage for specific phenotypes (Fig. 4). Identifying causal links for phage-specific effects in dysbiotic microbiota could light a path for therapeutic treatment. While pinpointing disease-associated signals in the phageome remains difficult, it seems that healthy individuals do have more overlap in their gut phage communities than diseased individuals, and that certain autoimmune and metabolic disorders are associated with alterations of the phageome or disease-specific phage contigs. Whether these disease-associated phageome changes are separable from shifts in bacterial hosts, whether one precedes the other, and whether either are causative or correlative, remain open and intriguing questions.

One exciting method of studying the causal effects of phage in the gut employs fecal microbiota transplants (FMT). FMT typically involves the transfer of fecal matter from a healthy donor to a diseased recipient, with the goal of restoring a robust and diverse microbiota in the recipient (48). FMT has been proven as an effective tool in the treatment of *Clostridioides difficile* infection (CDI) (49). While FMT samples usually contain both microbial and acellular components of the donor sample, it has been shown that cell-free fecal samples can also alleviate CDI symptoms, implicating a potential role for gut bacterial metabolites or bacteriophages in disease relief (50). Recent work has shown that bacteriophages from FMT donors can persist in their recipients for at least a year (51) and that successful engraftment of donor Caudoviricetes is associated with FMT success (52). More recently in mice, specific transfer of the fecal virome (FVT) has been shown to alleviate symptoms of type II diabetes, reduce small-intestine bacterial overgrowth, and promote healthy stress responses (53–55). These transferred bacteriophages could be acting therapeutically by pruning populations of harmful gut microbes, imposing diversifying selection, or by non-lytically altering resident hosts (Fig. 1). Transferred phages may also exert effects through direct interaction with the mammalian immune system. Studies of the *Pf* phage in *Pseudomonas aeruginosa* found that released *Pf* virions stimulate mammalian type-1 IFN pathways, which antagonize antibacterial TNF signaling and allow persistent infection by *Pf* lysogens (56). This and related findings provide evidence of direct interactions between phage particles and the mammalian immune system (57) (Fig. 2). These findings highlight the exciting possibility that with further research, exogenous phages could be used to re-shape a malleable microbiota, or directly interface with the human immune system in a therapeutic manner. In sum, many aspects of human health are tightly linked to the composition of gut phage communities. Answering the questions of (i) how the phageome causally affects disease, (ii) what therapeutic targets of the phageome look like, and (iii) how to reconfigure gut phage communities will require mechanistic dissection.

## DYNAMICS OF PHAGE–BACTERIA INTERACTIONS IN THE GUT

While the engineering of human gut phageomes and microbiotas is an appealing avenue of therapeutic intervention, at present, this pursuit is limited by a narrow understanding of how phage and bacteria interact in the gut. Much of the data regarding interactions of phage and bacteria in natural settings comes from examining communities in the ocean. Thus, comparative analysis of phage–bacteria interactions in the gut can only be performed robustly against communities in the ocean, despite stark differences in these environments. Recent findings suggest that the ecological dynamics of phages in the gut differ greatly from those in ocean environments. One key difference is the ratio between phage and host. This ratio, termed VMR (virus-to-microbe ratio) is typically high in the oceans, ranging from 3 to 161 phages for each bacterium, but remains around 1 to 1 in the gut (58–60). The VMR in oceans varies over time due to seasonal blooms of bacteria, while VMR in the gut remains stable (26, 61). In the oceans, warm seasons lead to increased nutrient availability and a bloom of prey bacteria. In response, prophages reactivate and lytically replicate, leading to a crash of the bacterial population and high VMRs (61, 62). In the gut, VMR remains low and varies minimally despite analogous post-feeding bacterial blooms (63). This implies a uniquely stable relationship between gut phages, their hosts, and the environment of the human gastrointestinal tract.

**Fig 3:**
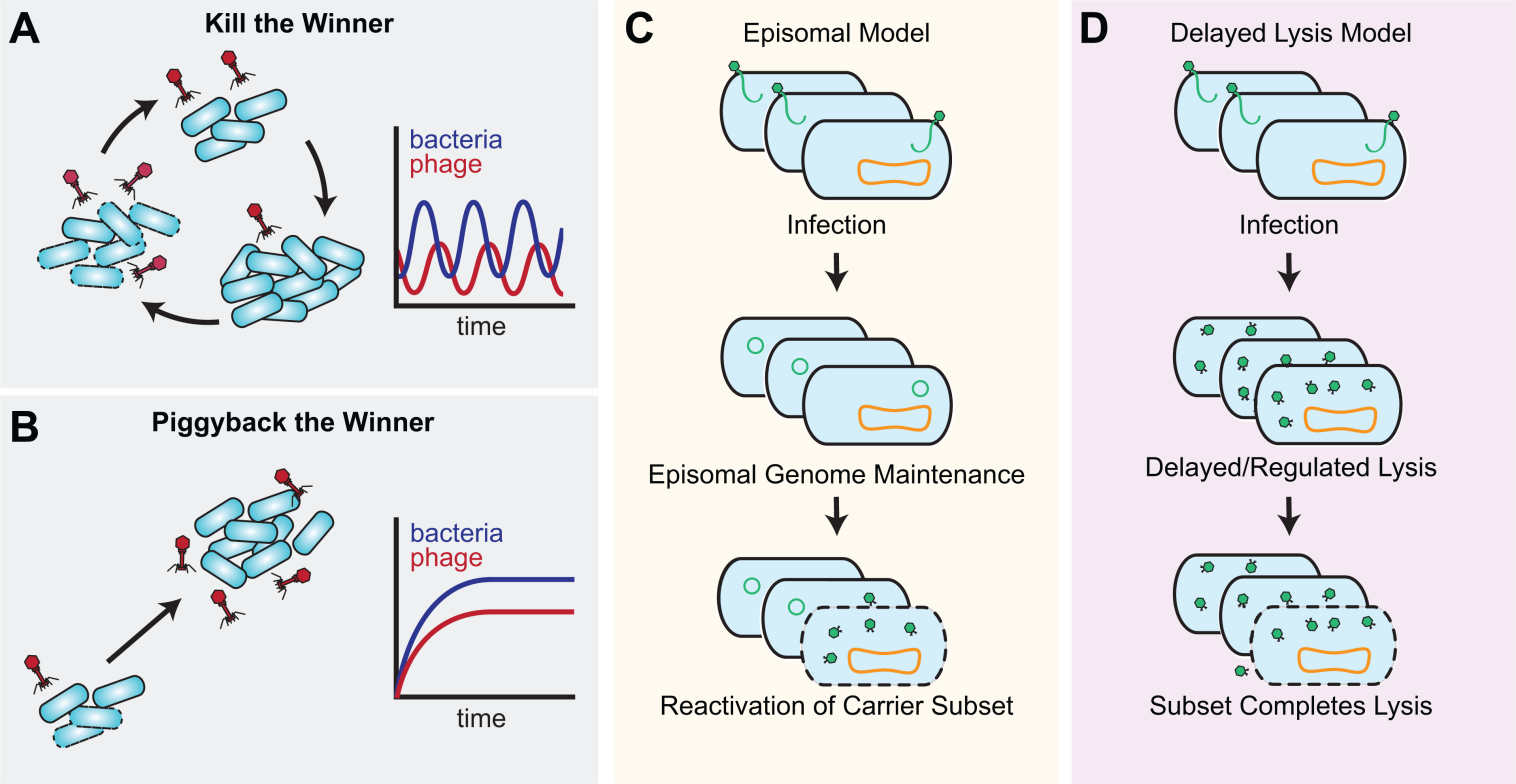
Frameworks for phage–host interactions. (**A**) In the kill-the-winner ecological framework, phages prey on the fastest-growing clones. Following a lytic epidemic, phage populations decay until the next clonal takeover. This results in offset oscillations of phage and host abundance. (**B**) In the piggyback-the-winner ecological framework, phage populations grow steadily as bacterial populations grow, potentially avoiding lytic epidemics through lysogeny, pseudolysogeny, or other low-VMR-type replicative strategies. In the absence of temperate representatives, how crAssphages and other virulent gut phages maintain low levels of stable replication remains a mystery. (**C**) One model to explain low VMR during crAssphage infection is that crAssphages remain in an episomal state until reactivating stimuli induce lytic replication. (**D**) Another model is that crAssphages have an extended and highly regulated lytic cycle.

The ecological framework that has been used to contextualize this stable association of gut phages with their hosts is the “piggyback the winner” (PtW) theory of phage–bacteria interactions (64). PtW takes its name from the more traditional “kill the winner” (KtW) model of predator–prey dynamics. In the KtW paradigm, phages kill the fastest-growing bacterial clones, promoting community diversity by limiting clonal takeover (65) (Fig. 3A). The KtW model is consistent with the observations of Stewart and Levin, who demonstrated that phage predation correlated positively to host *E. coli* growth rate (66). Stewart and Levin also proposed the corollary “hard times” hypothesis, wherein temperate bacteriophages remain dormant in their hosts when bacterial densities are too low to support lytic growth (67). The KtW and corollary “hard times” frameworks have a history of predictive accuracy in many planktonic environments (68, 69). The PtW framework bucks these ideas by proposing that phages remain non-lytic in times of high host density to piggyback off host growth success (Fig. 3B) (70). This contrasts with linear density-dependent predation models. The PtW concept was built off observations from environmental metagenomics that demonstrated decreases in VMR at the highest microbial densities. Both KtW and PtW have experimental support, and their application likely differs based on environmental context, host density, and the host–phage pair (64, 66). All considered, the low VMR and absence of phage lytic sweeps seen in sequenced phageomes reinforce the idea that PtW dynamics may govern phage–bacteria interactions in the gut.

One gut phage that embodies this unique low VMR replication strategy is the *Crassviridae* representative ΦCrAss001, which is one of the few crAssphages isolated in pure culture. ΦCrAss001 replicates with its bacterial host *Bacteroides intestinalis* at equilibrium VMRs of <1 in culture and in the mammalian gut (42). While these phages are thought to be virulent, their lysis might be delayed or only proceed in a small subset of cells (Fig. 3C and D). These lytic events undoubtedly have negative effects on individual hosts, but ΦCrAss001 does not affect bacterial population stability. Another crAssphage isolate recently reported to exhibit non-lytic replication in culture is *Carjvirus communis*, which adopts a phage-plasmid hybrid lifestyle (Box 1). The ability of *Crassviridae* to enter a tempered form of lytic replication is unique and fits in the broader characterization of gut phages as stably associating with their hosts. The extent of this relationship between crAssphages and their hosts, and the mechanism underlying the decision to replicate lytically or non-lytically await the isolation and characterization of additional representatives.

## SPATIAL DISTRIBUTION OF PHAGES AND BACTERIA IN THE GUT

Whether the ecological dynamics of phage–bacteria interactions are uniformly distributed throughout the gastrointestinal tract is an open question. The intestinal tract is a highly structured environment, which varies in conditions along the proximal-distal axis and radially from lumen to mucosa. A recent study using specialized sampling devices in humans emphasized how pH, bile acids, and metabolites vary along the length of the intestine (71, 72). These differences could contribute to a heterogenous distribution of bacteria and phages along the intestine’s length. Understanding the distribution of phages and their dynamics throughout the gut will be crucial in the design of targeted phage therapies. Using these specialized sampling devices, researchers found that gut phageome composition is similar between samples taken from stool, small, and large intestine. In fact, the intestinal and stool phage communities from one individual are more like each other (mean Jaccard distance 0.4) than the intestinal (0.62) or stool (0.58) phageomes between individuals. This paper also reported approximately threefold higher rates of prophage induction in proximal intestine compared to fecal samples. The rate of prophage induction correlated with the higher pH of the intestine, a phenomenon previously described for *E. coli* phages in the bladder (73). An abundance of induced prophages in stool has been associated with IBD but may be part of a healthy microbiota in the upper intestine (32).

Extensive work cataloguing gut phages along the intestine of *Rhesus macaques* found a somewhat uniform distribution in phage species but large differences in phage abundances along the intestine. Generally, the large intestine is more abundant in phage and uniquely includes members of *Crassviridae* and *Leviviridae* families (74). The authors also looked at the radial distribution of phages in the inner lumen and outer mucosa of macaques. They found, on average, one log fewer viral genome copies per gram of tissue in the intestinal mucosa compared to the lumen. This is in accordance with data from recent work that found three logs fewer plaque-forming units in the intestinal mucus of mice than their intestinal lumens post-phage oral gavage (75). The paucity of phages in the intestinal mucosa mirrors that of their bacterial hosts, which are far more abundant in the lumen. The former authors postulated that an absence of phages in the intestinal mucosa could create a spatial refuge for phage-sensitive bacteria (74). Opposing this idea, previous work has shown that certain phages bind to and accumulate in intestinal mucus layers (76). The adherence of *E. coli* phages to mucus enhances the ability of phage to clear invasive pathogens from the mucosa. This concept of phage-derived immunity to invasive pathogens has been coined the bacteriophage adherence to mucus (BAM) model. The spatial refuge and BAM model are not necessarily exclusive, as the BAM model may only apply to specific or sparse phage–host pairs, allowing other hosts a refuge in the mucosa. Synthesizing the data on spatial distribution of phages in the gut, Shkoporov et al. posit that PtW dynamics dominate in the crowded lumen, where the VMR is low, while classical KtW paradigms are more common where phages are sparse in the mucosal layers (4). Adding to this, the distribution of phages in the lumen or mucosa could vary during periods of feeding or fasting, which would have interesting consequences for BAM-mediated immunity. Taken together, it seems that phages are more consistently distributed along the proximal-distal axis than the radial axis, as are the high-level dynamics of phage–bacteria interactions.

## ANTAGONISTIC CO-EVOLUTION OF GUT PHAGES AND THEIR HOSTS

With the predominance of stable host–phage associations in the gut, it may seem that gut phages and their hosts are in a para-symbiotic relationship. Yet, there is abundant evidence of genetic conflict. To start, bacteria in the gut harbor similar anti-phage immune systems in comparable abundances as their counterparts in the soil, despite stark differences in VMR (77). Among the abundant conserved immune systems are restriction-modification (RM) systems, CRISPR-Cas systems, and many that are classified as abortive-infection (Abi) systems. Bioinformatic analysis of these CRISPR-Cas systems, which store targeted viral sequences as “spacers” in CRISPR arrays, can reveal much about phage–host interactions. Phage-derived spacers, once integrated, serve as an immunological record of infection. As acquisition of new spacers is rare, and spacers can be lost through recombination over time, spacer analysis does not provide a comprehensive record of all phages encountered by that host but does provide a snapshot of a set of historical interactions. By analyzing the content and abundances of bacterial CRISPR arrays along with their target phages from microbiota samples, López-Beltrán et al. were able to track antagonistic interactions of gut phages and their hosts (78). One of their key findings was the high conservation of spacers targeting prophages and crAssphages. Of the 8,881 mapped spacers, 43% targeted temperate phages. This could be explained by the abundance and success of these phages in the gut, as well as the longer residence times of these phage types, which could allow increased spacer acquisition. The signature of prophage-targeting spacers has also been seen in a study of *E. coli* colonizers of the infant gut, where 100% of mapped spacers target temperate phages (79). These numbers may be specifically influenced by the temperate phage dominant character of the infant gut. A plurality of temperate phage targeting spacers is surprising given that CRISPR targeting of temperate phages or an integrated prophage can lead to genome damage, cell death, and loss of the CRISPR system (80, 81). While only 10% of these spacers were truly confirmed as “self-targeting” in the latter study (i.e., a target in the same chromosome as the spacer), targeting of non-integrated temperate phages in the population can still lead to genome damage and CRISPR deletion. Either way, it must be critical for early colonizers to avoid the lethality of self-targeting while invading a new niche. Thus, the prevalence of prophage targeting spacers implies a segregation of lysogen and spacer harboring sub-populations, or the presence of anti-CRISPR (Acr) mechanisms to repress CRISPR systems during lysogeny (82, 83). Indeed, phage-encoded anti-CRISPR proteins have been discovered in the prophages of several gut microbes and could serve to enable lysogeny despite spacer acquisition (84, 85). In any case, accumulation of temperate phage targeting spacers in the face of potential self-targeting highlights the strong selective pressures temperate phages place upon their hosts in the gut. This upends the concept that prophages are mostly quiescent in the gut. Combining these findings with the increased rate of prophage induction seen in the upper intestine implies that temperate phages may play an unappreciated role in the intestinal microbiota. Furthermore, by tracking relative abundances of spacers and their target phages, López-Beltrán et al. were able to show that following acquisition of a new spacer, targeted phage populations decline sharply but do not go extinct (78). On the host side, phage predatory sweeps led to weak selection of hosts containing a cognate spacer. While these successive selective sweeps may have a small amplitude, and not be evident in total VMR measurements, they are indicative of arms race or fluctuating selection dynamics. Whether these dynamics apply for other gut bacterial immune systems remains to be seen.

Antagonistic co-evolutionary dynamics of gut phages and their hosts also exist outside of bacterial immune systems, at the barrier to phage entry. Many gut *Bacteroides* species encode phase-variable capsular polysaccharides (CPS) genes, which use inverting promoter sequences to allow heterogenous expression of CPS variants (86). Certain CPS variants allow *Bacteroides* to evade host immune clearance in the gut, and the ability to switch between CPS variants promotes antibiotic tolerance (87). Only recently has it been appreciated that CPS is a key determinant of phage resistance or susceptibility (40, 42). CPS variants may affect host susceptibility by acting as a phage receptor, by shielding a receptor, or otherwise affecting phage binding. A 2020 paper by Porter et al. (40) isolated 71 *Bacteroides* phages by plating fecal filtrates on lawns of wild-type *Bacteroides* or mutants expressing any one or none of the eight possible CPS variants. Of the 71 phages isolated, 40.8% preferred an acapsular host, and the remaining preferred a unique CPS variant. These data argue that in some cases, CPS aids in phage infection, and in others, hinders it. Thus, a population of *Bacteroides* with heterogenous CPS variants can adapt to the selective pressures of different phages, the pressures of the host immune system, and the environmental stressors. The paper also demonstrates the importance of examining cultured phage–host pairs, as computational predictions of these phage–host binding interactions remain limited. On the phage side of the conflict, a large portion of intestinal phages encode diversity-generating retroelements (DGRs), which allow reverse transcriptase-mediated variation of a genetic locus. In a metagenomic analysis of almost 200,000 intestinal phages, DGR-associated reverse transcriptases were the third-most abundant functionally annotated protein, supplanted only by phage integrases and the helix-turn-helix DNA-binding domain (88). These DGRs were present along with a targeted hypervariable locus in 84% of intestinal Myoviruses. Among the most prevalent phage genes in these hypervariable regions are Ig-like domains and tail-collar fiber proteins, which play a role in attachment to bacterial hosts. Hypervariation of host-binding structures may allow phage to adapt to ever-changing bacterial attachment factors or could facilitate the evolution of generalist phages, which jump from one host to another.

## PHAGE–BACTERIA MUTUALISM IN THE GUT

It has been argued that the phase variability of *Bacteroides* CPS is not a strategy for phage restriction but rather a strategy to maintain a stable phage population through retention of both sensitive and resistant host populations (42). Maintenance of a phage population could benefit bacterial hosts through a variety of mechanisms, including lysogenic conversion, competitor inhibition, horizontal gene transfer (HGT), and phage-driven diversification of host bacteria. During lysogenic conversion by temperate phages, prophage-encoded genes can increase fitness of their hosts. One example of this in the gut is the *bxa* prophages of *Bacteroides*, which encode ADP ribosyltransferases (ADPRTs) that are secreted during gut colonization. These ADPRTs affect epithelial cell metabolism, inducing the production and secretion of inosine. *Bacteroides* can utilize this secreted inosine as a carbon source, enhancing intestinal colonization (89).

Another way temperate phages can benefit their hosts is through lysis of competitor bacteria. Lysogenic populations experience a constant low level of prophage induction (~1% of cells), which produces phage particles that will not replicate in the lysogenic population but will kill closely related non-lysogens (90). While the induction comes at the cost of host lysis, the killing of sensitive competitors can result in net fitness gains for the lysogenic population (91). Furthermore, the phage particles released from sensitive competitor cells can transfer novel genetic material back to the original lysogenic population in a process termed “autotransduction” (92). This and other forms of phage-mediated HGT may provide benefit to their bacterial hosts through the spread of adaptive alleles. Despite a plethora of mechanisms, quantifying the rate and range of phage-mediated HGT in the gut remains difficult. Broadly, HGT can be tracked through phylogenetic analysis or by looking for bacterial DNA in phage capsids. Phylogenetic analysis can detect transfer of novel genes into recipient cells by temperate phages but poses many technical challenges and has limited ability for quantification (93). Looking for bacterial DNA in phage capsids can be more quantitative, but there are difficulties in completely separating phage capsids from contaminating bacterial DNA (94). The approach of aligning phage-packaged DNA to bacterial genomes was termed “transductomics” and has been used to track transduction in the mouse intestinal microbiota. A similar approach was employed by Modi et al. in 2013, who found that phageome extractions from mice treated with antibiotics were enriched in antibiotic resistance genes from a bacterial origin (95). This finding suggested that phages help mobilize antibiotic resistance genes following antibiotic treatment. Notably, a subsequent reanalysis of the data from Modi et al. concluded that all bacterial gene clusters were enriched in phageome sequences after antibiotic treatment, with no selection for antibiotic resistance genes (96). A recent paper using transductomics-like approaches paired with long-read sequencing identified large insertions of plasmid and bacterial DNA into gut phage genomes, extending the identifiable events of transduction in the gut (97). These transductomics approaches are becoming more feasible but have yet to be used in fully characterizing a network of phage-mediated HGT in the human gut. With improved purification, sequencing, and analysis methods, identifying the full network of phage-mobilized genes and their contribution to bacterial antibiotic resistance and other clinically relevant phenotypes lies within reach.

Characterizing finer ecological dynamics of gut phages and their hosts will eventually require adoption of experimental model systems. Model phageome and bacteriome communities would allow quantification of mutualistic and antagonistic dynamics as well as the effects of disease states or environmental perturbations. A complementary approach to answering these evolutionary questions is longitudinal sampling. Longitudinal sampling and time-shift challenge assays have been instrumental in characterizing phage–bacteria interactions between *Vibrio* and their phages (98). These experiments have identified fluctuating selection dynamics and unique relationships between phage predation and antibiotic resistance mobility. While the healthy gut microbiota is a complex and crowded environment, time-shift experiments could illuminate evolutionary dynamics in keystone microbes and complement the foundation of metagenomic studies. In sum, while gut phages and their hosts achieve a stable equilibrium at uniquely low VMRs, and extinction events remain rare, underneath this stability is constant genetic conflict, mutualism, and exchange. Why gut phages equilibrate at such low VMRs in the gut thus remains an open question. Perhaps the unique structure and density of the gut microbial community alone promote diversification and prevent large phage sweeps through clonal populations (99). This general feature of the gut might also explain why phage–host stability and low VMR prevail among a range of gut phages and even archaeal viruses (100). Whether the low VMR equilibrium of the gut could be disrupted by nutrient fluctuation or other perturbations would be an interesting experimental vein. To answer these and other questions regarding the stability of phage–bacterial interactions in the gut will require a synergy of powerful experimental models along with ever-expanding metagenomics tools.

## EXPERIMENTAL MODELS FOR STUDY OF THE PHAGEOME

One significant hurdle to developing experimental models and dissecting phage–bacterial interactions in the gut is the difficulty in identifying phage–host pairs. There have been numerous *in silico* approaches developed recently to characterize phage–host pairs. These methods utilize sequence similarity or machine learning models to make predictions about receptor-ligand binding, prophage residence, and protein–protein interactions of co-isolated phages (101–112). One simple bioinformatic method for identifying phage–host pairs is to analyze CRISPR spacer content (113–115). Another approach is metagenomic high-throughput chromosome conformation capture (metaHi-C), which uses chemical cross-linking to physically join co-habitating phage and bacterial genomes. These linked genomes are then ligated and sequenced together, giving high-resolution information about the chromosomal interactions of phage and host. The advantage of this approach is that it captures phage genome dynamics within the host cell (60, 116, 117); however, it does not detect historical associations like the CRISPR spacer analysis methods. Alternatively, a single-cell viral tagging can be used to identify phage–host pairs by unspecified fluorescent labeling of viruses, which bind and adsorb to prokaryotic hosts that can then be sorted and sequenced individually (118). Identification of phage–host interactions in native gut microbiota will allow for the engineering and assessment of representative gut phage communities, which can then be dissected in model host systems.

**Fig 4:**
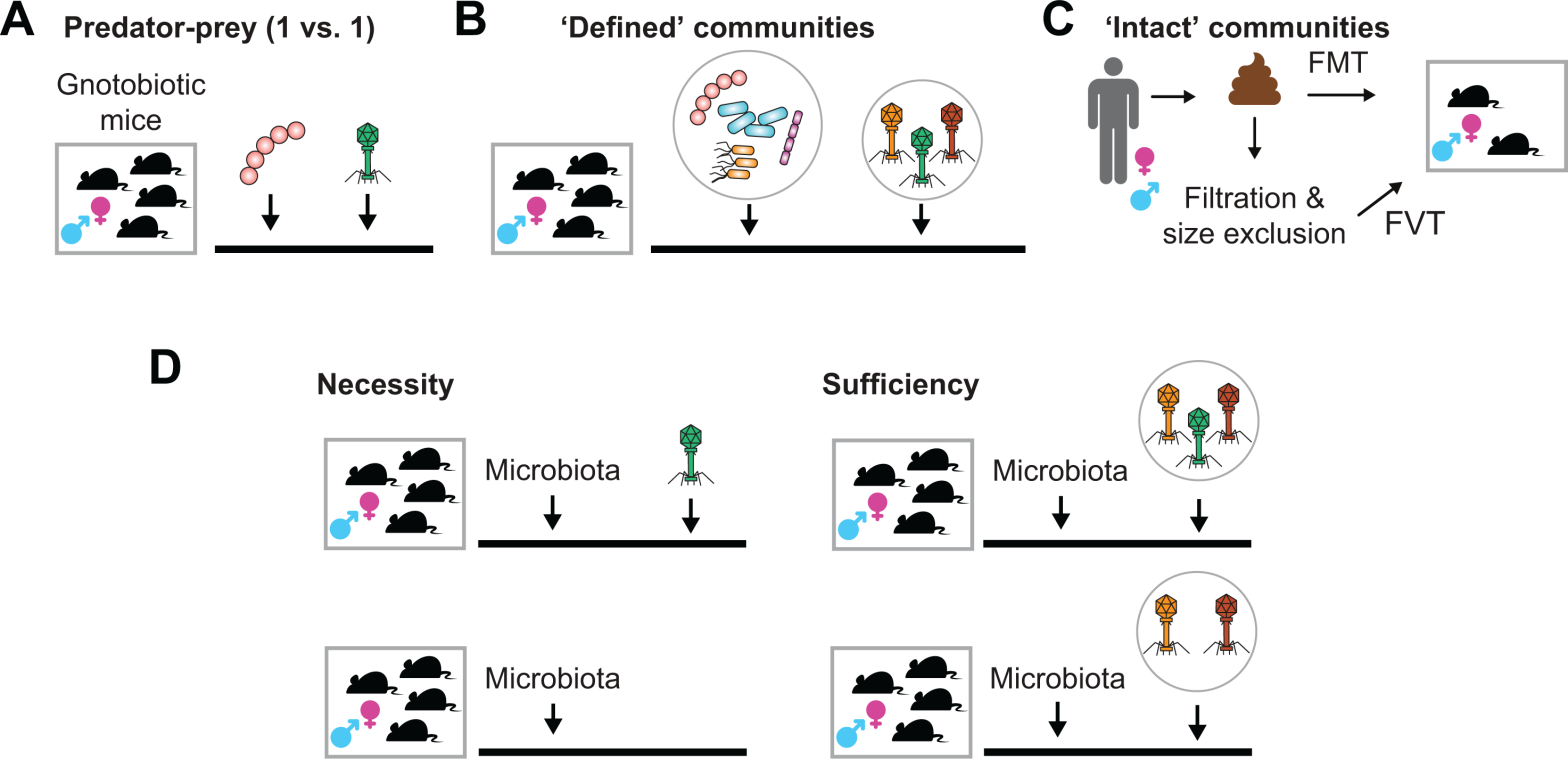
*In vivo* experiments to examine causal roles for phage in bacterial and host phenotypes. (**A–C**) The effects of phage on their bacterial targets can be examined in gnotobiotic animals colonized with single isolates (**A**), communities with “defined” inocula (**B**), or complex and undefined “intact” communities via fecal microbiota transplants (FMTs) and fecal virome transplants (FVTs) (**C**). (**D**) The sufficiency of a phage isolate for a host phenotype can be assessed by using a monocolonized arm of animals (top left experimental group) compared to uncolonized controls (bottom left group). The necessity of the isolate for a host phenotype can be examined by comparing a complete cocktail of phages (top right experimental group) compared to one in which it is excluded from the cocktail (bottom right group).

Advances in cell- and animal-based models have the potential to uncover causal links between the life cycle of gut phages and microbiota homeostasis. Cell-based “gut-on-a-chip” models use micrometer channels to allow the continuous flow of media, which can remove dead cells and other cellular metabolites, milieu, and allow phage particle efflux. These microfluidic platforms simulate the intestinal environment and have been able to recapitulate *in vivo* phenotypes of phage–host predation at the mucosal surface (119–122). Gnotobiotic (i.e., germ-free) mouse models have laid the groundwork for the attribution of causal roles for specific bacterial strains in various contexts. These mice are raised in a sterile environment devoid of microbes to then be colonized with “defined” communities of microbes. Phages can then be introduced as isolates, cocktails of specific phages, or entire phage communities as FVT (Fig. 4A through C). Using intricate experimental designs, gnotobiotic animals can be used to determine whether single phage isolates are necessary and/or sufficient for observed biological effects or desired community engineering efforts (Fig. 4D). Mock phage communities delivered to gnotobiotic animals by FVT have shown that phages can have a significant effect on bacterial community structure and that resident prophages protect hosts from transplanted phage predation (123). Furthermore, FVTs can be treated to inactivate different viral strata such as eukaryotic, enveloped, or RNA viruses within the virome (124). These experimental models allow researchers to identify causal roles for phage in microbiome homeostasis.

## CONCLUDING REMARKS

Akin to the early days of microbiota research, a lot has been learned in recent decades about the gut phageome through the accounting of community members, their abundances, and their distributions in different individuals. While viral “dark matter” remains abundant and uncharacterized, our mechanistic understanding of gut phage–host interactions lags far behind our understanding of gut phageome composition and associations with lifestyles and disease. Advances in isolation and culturing techniques along with a broader use of experimental models will advance mechanistic understanding of these interactions. Open areas of inquiry in the field include (i) the causal links between gut phages and states of disease or homeostatic processes, (ii) the factors underlying stable association of gut phages with their hosts, and (iii) how phages can be used to engineer the microbiota. These questions along with the endless potential for novel phage biology in the microbiota will doubtlessly drive a generation of phage biologists into the gut.
